# Association Between Hypertensive Disorders of Pregnancy and Patent Ductus Arteriosus in Very Preterm Infants: A Bayesian Model-Averaged Meta-Analysis

**DOI:** 10.3390/children12060762

**Published:** 2025-06-12

**Authors:** Moreyba Borges-Luján, Gloria Galán-Henríquez, Rosa I. Rodríguez-Viera, František Bartoš, Gema E. González-Luis, Eduardo Villamor

**Affiliations:** 1 Department of Pediatrics, Hospital Universitario Materno-Infantil de Canarias, 35016 Las Palmas de Gran Canaria, Spain; 2 Department of Psychology, University of Amsterdam, 1018WS Amsterdam, The Netherlands; 3 Division of Neonatology, MosaKids Children’s Hospital, Maastricht University Medical Center (MUMC+), Research Institute for Oncology and Reproduction (GROW), Maastricht University, 6202AZ Maastricht, The Netherlands

**Keywords:** patent ductus arteriosus, preeclampsia, preterm infant, Bayesian statistics, meta-analysis

## Abstract

**Background/Objectives**: Prenatal adverse events may influence the development of complications of prematurity, including patent ductus arteriosus (PDA). We conducted a systematic review and Bayesian model-averaged (BMA) meta-analysis of observational studies exploring the association between hypertensive disorders of pregnancy (HDP) and the risk of PDA in preterm infants. **Methods**: PubMed/Medline and Embase databases were searched. We used BMA analysis to calculate Bayes factors (BFs). The BF_10_ is the ratio of the probability of the data under the alternative hypothesis (H_1_, presence of association) over the probability of the data under the null hypothesis (H_0_, absence of association). **Results**: We included 41 studies (58,004 infants). BMA analysis showed moderate evidence in favour of H_0_ for the association between HDP and any PDA (BF_10_ = 0.20) as well as for the association between HDP and hemodynamically significant PDA (BF_10_ = 0.27). Subgroup analyses based on the subtype of HDP showed that the moderate evidence in favour of H_0_ was only conclusive (i.e., BF_10_ < 0.33) for the associations of any PDA with preeclampsia (BF_10_ = 0.30) and hemodynamically significant PDA with preeclampsia (BF_10_ = 0.17). **Conclusions**: The currently available evidence suggests a lack of association between HDP and the risk of developing PDA.

## 1. Introduction

The closure of the ductus arteriosus (DA) at birth is a central event in the transition from foetal to neonatal circulation [1,2,3,4]. In very and extremely preterm infants (i.e., gestational age < 32 weeks), ductal closure is frequently delayed or fails, resulting in a clinical situation referred to as patent ductus arteriosus (PDA). Diagnosing and managing PDA is a key part of the neonatal intensive care practice [1,2,3,4].

Precision or personalized medicine is becoming an overarching medical discipline that requires a better understanding of genotypes, endotypes, and clinical phenotypes of diseases [5]. The term endotype refers to “a subtype of a condition, which is defined by a distinct functional or pathophysiological mechanism” [6]. Despite the absence of a definitive consensus on the identification of endotypes associated with prematurity, the integration of clinical data with placental and bacteriological information allows for the delineation of two predominant pathways leading to very and extremely preterm birth: infection/inflammation and dysfunctional placentation [7,8,9]. The first group includes chorioamnionitis, pre-labour premature rupture of the membranes, and cervical insufficiency [7,8,9]. The second group is defined by the presence of histologic features indicative of placental vascular dysfunction. This group is associated with hypertensive disorders of pregnancy (HDP) and the condition is referred to as foetal indication/intrauterine growth restriction (IUGR) [7,8,9]. The environment to which the developing foetus is exposed varies greatly between the two endotypes of prematurity. In the infectious endotype, the response is inflammatory, with a release of cytokines and other inflammatory mediators, whereas in the placental dysfunction endotype, there is chronic hypoxia and imbalance of angiogenic mediators [7,8,9]. Therefore, the different endotypes may have a variable potential to induce the different complications of prematurity [7,8,9].

Our group has recently analysed the association between the different endotypes of prematurity and its major complications, including PDA [10,11,12,13]. We observed a statistically significant (*p* < 0.001) association between chorioamnionitis and the risk of developing PDA [10]. The odds ratio (OR) was 1.35 with a 95% confidence interval (CI) of 1.17 to 1.56. However, this association was strongly related to the lower gestational age (GA) of infants exposed to chorioamnionitis compared to those not exposed [10]. Regarding the endotype of placental dysfunction, we examined the association between PDA and IUGR in another meta-analysis [13]. We found a statistically significant association (OR 0.82, 95% CI 0.70 to 0.96, *p* = 0.015) between growth restriction (defined as IUGR or small for GA) and the risk of developing PDA. However, this statistical significance was not present (OR 0.87, 95% CI 0.72 to 1.04, *p* = 0.133) when only hemodynamically significant PDA (hsPDA) was taken into account [13].

HDP, including preeclampsia, eclampsia, and gestational hypertension, are among the most common causes of preterm birth and are often associated with the various complications of prematurity [14]. As mentioned above, HDP are among the conditions considered to be part of the endotype of placental dysfunction [12]. Of note, the potential association between HDP and PDA has not been the subject of extensive investigation. In a meta-analysis published in 2021, Liu et al. examined the relationship between various antenatal conditions and PDA [15]. They included eight studies that reported on rates of preeclampsia and PDA and found no statistically significant association between the two conditions (OR 0.89, 95% CI 0.68–1.17, *p* = 0.406). Our objective in the present study was to perform a systematic review and meta-analysis of the potential association between HDP and the risk of developing PDA. Instead of the more commonly utilized frequentist statistics, including *p*-values and confidence intervals, a Bayesian approach was used for the meta-analysis. A key limitation of frequentist null hypothesis significance testing is that failing to reject the null hypothesis (H_0_) when the *p*-value is below a predetermined threshold (typically 0.05) or the confidence interval intersects the line of no effect does not necessarily indicate evidence for H_0_ [16]. The Bayesian approach allows for the assessment of the strength of the evidence in relation to both H_0_ (absence of effect/association) and the alternative hypothesis (H_1_, presence of effect/association) [16].

## 2. Materials and Methods

This study was performed and reported according to the preferred reporting items for systematic reviews and meta-analyses of observational studies in epidemiology guidelines. Review protocol was registered in PROSPERO database (ID = CRD42020184843). The Population, Exposure, Comparison, and Outcome (PECO) question was: Do preterm infants (P) exposed to HDP (E) have a higher risk of developing PDA (O) than preterm infants with no history of exposure (C)?

*Sources and search strategy*

A comprehensive literature search was undertaken using the PubMed and Embase databases. The search strategy is detailed in Supplementary Table S1. No language limit was applied. The literature search was updated up to February 2024.

*Study selection and definitions*

Studies were included if they had a prospective or retrospective cohort or case-control design, examined very and extremely preterm infants (GA ≤ 32 weeks), and reported primary data that could be used to measure the association between exposure to HDP and PDA. The reason for focusing on very and extremely preterm infants was that the risk of PDA increases as GA decreases [1,2,3,4]. The studies were divided according to the way they considered small ductal shunts. Studies comparing closed DA vs. small plus large PDA were classified as reporting on “any PDA”. Studies comparing closed DA and/or small PDA vs. large PDA were classified as reporting on “hemodynamically significant PDA” (hsPDA). In the absence of a universally accepted definition of hsPDA, any definition included in the original studies was accepted for the purposes of this analysis. PDA requiring treatment was considered a proxy for hsPDA. The HDP category was further subdivided into preeclampsia, preeclampsia/HELLP (Hemolysis, Elevated Liver enzymes and Low Platelets) syndrome, and “any HDP”. As with PDA, any definition of HDP, preeclampsia, or HELLP syndrome provided by the original studies was accepted for the purposes of this analysis.

To identify relevant studies, two reviewers (GGL and EV) independently screened the results of the searches and applied inclusion criteria using a structured form. Discrepancies were identified and resolved through discussion or in consultation with the other researchers.

*Data extraction and assessment of risk of bias*

Three investigators (MB-L, GG-H, and RR-V) extracted data on the study design, demographics, and rate of PDA. A second group of investigators (GG-L and EV) checked the data extraction for completeness and accuracy. Risk of bias was assessed using the Newcastle–Ottawa scale (NOS) for cohort or case-control studies [17]. NOS scores ≥ 7 were considered indicative of low risk of bias and scores of 5 to 6 as indicative of moderate risk of bias.

*Bayesian model-averaged meta-analysis*

The effect size of dichotomous variables was expressed as logOR and the effect size of continuous variables was expressed using the Hedges’ *g*. Values of logOR and Hedges’ *g* and the corresponding standard error of each individual study were pooled and analysed by a Bayesian model-averaged (BMA) meta-analysis [18,19]. We performed the BMA analysis in JASP, which utilizes the RoBMA R package [20]. BMA analysis employs Bayes factors (BFs) and Bayesian model-averaging to evaluate the likelihood of the data under the combination of models assuming the presence vs. the absence of the meta-analytic effect and heterogeneity [18,19]. The BF_10_ is the ratio of the probability of the data under H_1_ over the probability of the data under H_0_. The BF_10_ was interpreted using the evidence categories suggested by Lee and Wagenmakers [21]: <1/100 = extreme evidence for H_0_, from 1/100 to <1/30 = very strong evidence for H_0_, from 1/30 to <1/10 = strong evidence for H_0_, from 1/10 to <1/3 = moderate evidence for H_0_, from 1/3 to <1 weak/inconclusive evidence for H_0_, from 1 to 3 = weak/inconclusive evidence for H_1_, from >3 to 10 = moderate evidence for H_1_, from >10 to 30 = strong evidence for H_1_, from >30 to 100 = very strong evidence for H_1_, and >100 extreme evidence for H_1_. The BF_rf_ is the ratio of the probability of the data under the random-effects model over the probability of the data under the fixed-effects model.

We used robust Bayesian meta-analysis (RoBMA) to assess the robustness of the results to the potential presence of publication bias, which was expressed as BF_bias_ [22]. The potential moderating effect of the difference in GA (the mean GA of the HDP-exposed group minus the mean GA of the non-exposed group in each individual study) on the association between HDP and PDA was investigated through Bayesian model-averaged meta-regression (BMA-reg) and expressed as BF_mod_ [23]. The categories of strength of the evidence in favour of the random effects (BF_rf_ > 1) or the fixed effect (BF_rf_ < 1), presence vs. absence of publication bias (BF_bias_ >1 vs. BF_bias_ <1), or presence vs. absence of moderation by GA (BF_mod_ > 1 vs. BF_mod_ < 1) were similar to those described above for the BF_10_.

For all the analyses, we used the neonatal-specific empirical prior distributions based on the Cochrane Database of Systematics Reviews [18]. Binary outcomes: logOR ~ Student-t (µ = 0, σ = 0.29, ν = 3), tau (logRR) ~ Inverse-Gamma (k = 1.80, θ = 0.42); Continuous outcomes: Cohen’s d~Student-t (µ = 0, σ = 0.42, ν = 5), tau (Cohen’s d)~Inverse-Gamma (k = 1.68, θ = 0.38) [18]. For the meta-regression, we used the prior distributions for the effect size and heterogeneity parameter with the prior distribution on the standardized meta-regression coefficient scaled by 1/2 (i.e., the expected difference in each moderator level corresponding to 1/2 of the mean effect size) [23]. To test the robustness of this analysis, another meta-regression was conducted with the prior distribution on the standardized meta-regression coefficient scaled by 1/4 (Supplementary Table S3).

## 3. Results

### 3.1. Description of Studies and Risk of Bias Assessment

**Figure 1:**
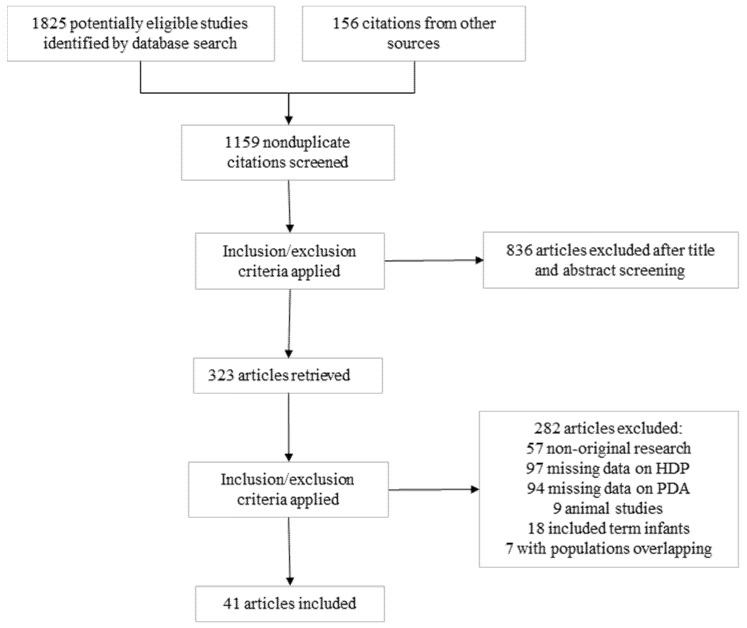
Flow diagram of the systematic search.

Of 1159 potentially relevant studies, 41 (58,004 infants) were included [24,25,26,27,28,29,30,31,32,33,34,35,36,37,38,39,40,41,42,43,44,45,46,47,48,49,50,51,52,53,54,55,56,57,58,59,60,61,62,63,64] (Figure 1). Their characteristics are summarized in Supplementary Table S2. Two studies were case-control studies and the other 39 were cohort studies. Sixteen studies were prospective and 25 were retrospective. Sixteen studies reported on any PDA, 24 reported on hsPDA, and one study reported on both any PDA and hsPDA. The quality score of each study according to the NOS is depicted in Supplementary Table S2. All studies received a score of six or higher, indicating a low to moderate risk of bias.

### 3.2. Bayesian Model-Averaged Meta-Analysis

**Figure 2:**
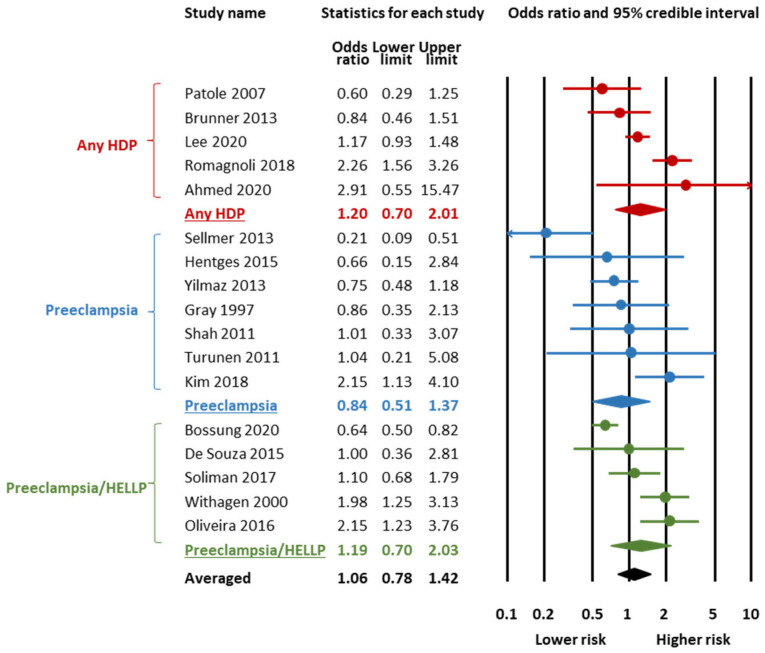
Bayesian model-averaged (BMA) meta-analysis of the association between hypertensive disorders of pregnancy and any patent ductus arteriosus in preterm infants [24,27,28,30,35,37,42,43,45,47,49,51,52,54,57,61,63].

**Figure 3:**
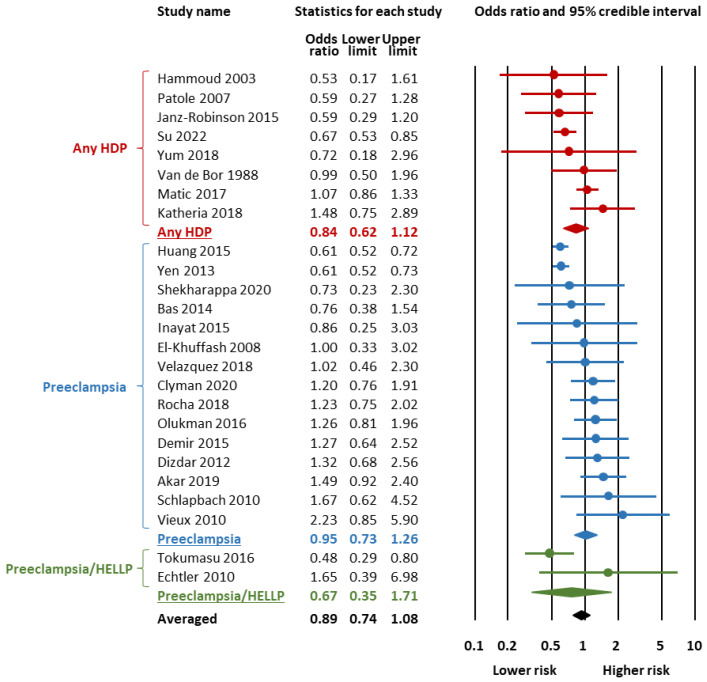
Bayesian model-averaged (BMA) meta-analysis of the association between hypertensive disorders of pregnancy and hemodynamically significant patent ductus arteriosus in preterm infants [25,26,29,31,32,33,34,36,38,39,40,41,44,46,47,48,50,53,55,56,58,59,60,62,64].

Figure 2 and Figure 3 and Table 1 summarize the results of the BMA meta-analysis. LogOR was converted to OR for clarity. BMA analysis showed moderate evidence in favour of H_0_ (absence of association) for the association between HDP and any PDA (BF_10_ = 0.20, Figure 2, Table 1) as well as for the association between HDP and hsPDA (BF_10_ = 0.27, Figure 3, Table 1). BMA analysis showed extreme evidence of heterogeneity (BF_rf_ > 100) for both any PDA and hsPDA (Table 1). For both meta-analyses, the RoBMA did not find evidence in favour of publication bias (Any PDA: BF_bias_ = 0.49; hsPDA: BF_bias_ = 0.33, Supplementary Table S3).

Sixteen studies reported the mean or median GA of infants exposed and unexposed to HDP during gestation [27,30,35,37,38,42,44,48,50,54,55,56,57,61,63]. Infants exposed to HDP had a GA at birth 0.97 weeks higher (Standard error = 0.16) than unexposed infants. This difference corresponds to a value of Hedges’ g of 0.44 (95% CrI 0.28 to 0.60). BMA analysis showed that the evidence in favour of this higher GA in the HDP-exposed group was extreme (BF_10_ = 2498).

### 3.3. Subgroup Analysis and Meta-Regression

Subgroup analyses based on the subtype of HDP showed that the moderate evidence in favour of H_0_ was only present for the associations of any PDA with preeclampsia (BF_10_ = 0.30, Table 1, Figure 2) and hsPDA with preeclampsia (BF_10_ = 0.17, Table 1, Figure 3). For the other HDP subtypes, the BMA analysis showed weak evidence in favour of H_0_ (Table 1).

**Figure 4:**
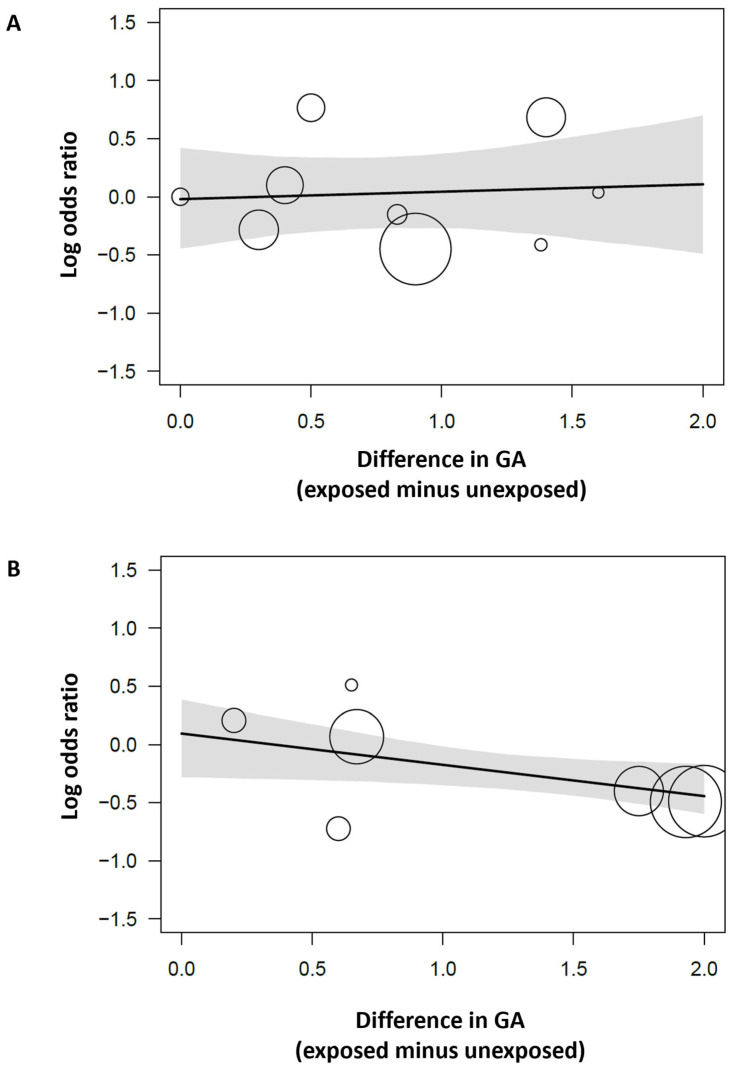
Bayesian model-averaged meta-regression (BMA-reg). Meta-regression plot showing the correlation between the effect size (log OR) of the association between hypertensive disorders of pregnancy and (**A**) any patent ductus arteriosus and (**B**) hemodynamically significant ductus arteriosus and the difference in gestational age (GA, exposed minus unexposed group in each individual study). The size of the circles is proportional to the contribution of each individual study to the association.

Meta-regression showed that the differences in GA between the infants exposed or unexposed to HDP in each individual study correlated with the effect size (logOR) of the association between HDP and hsPDA (seven studies; standardized meta-regression coefficient: −0.20, 95% CrI −0.34 to 0.02; BF_mod_ = 5.75), but not with the effect size of the association between HDP and any PDA (nine studies; meta-regression coefficient: 0.03, 95% CrI −0.20 to 0.26; BF_mod_ = 0.86) (Figure 4, Supplementary Table S4).

## 4. Discussion

To the best of our knowledge, this study represents the most comprehensive examination of the potential association between HDP and PDA in infants with GA *≤* 32 weeks. A broad search strategy has enabled the inclusion of a greater number of studies than those included in previous meta-analyses. Moreover, the application of a Bayesian approach has facilitated the drawing of more nuanced conclusions. A frequentist approach would have led to the conclusion that no statistically significant association was found between HDP and any PDA (*p* = 0.514) or hsPDA (*p* = 0.348). However, it would not have been possible to ascertain whether these findings provided evidence of no association (H_0_ should be accepted) or indicated an absence of evidence (the data are inconclusive to accept or reject H_0_). Bayesian hypothesis testing aims to quantify the relative plausibility of H_1_ and H_0_. Thus, the Bayesian approach allows us to estimate that the observed data are five times (BF_10_ = 0.20; BF_01_ = 5.00) more likely under no association (H_0_) between HDP and any PDA than under the presence of association (H_1_). Similarly, the observed data are 3.7 times (BF_10_ = 0.27; BF_01_ = 3.70) more likely under no association between HDP and hsPDA than under the presence of association. We can therefore conclude that there is moderate evidence for an absence of association between HDP and both any PDA and hsPDA.

Adverse prenatal conditions may have a profound effect in foetal development as well as in the clinical outcome of preterm infants. It is therefore biologically plausible that HDP may affect ductal development, resulting in an altered risk of PDA. Of note, a number of population-based studies have demonstrated an association between early-onset preeclampsia (<34 weeks of gestation) and congenital cardiac defects in offspring [65,66]. These defects may affect all general structures of the heart, including the aorta, pulmonary artery, valves, ventricles, and septa [65,66]. The proposed mechanism for this association is the effect on cardiovascular development of the imbalances in proangiogenic signalling proteins, such as vascular endothelial growth factor and placental growth factor, and antiangiogenic proteins, such as soluble endoglin and fms-like tyrosine kinase 1 [65,66,67,68]. This angiogenic imbalance may be involved in the pathophysiology of preeclampsia as well as in the pathogenesis of congenital heart defects [65,66,67,68]. Interestingly, PDA in term newborns is one of the conditions that has been associated with early-onset preeclampsia [65]. However, the etiopathogenic mechanisms underlying PDA in term and preterm newborns are distinct. In term and late-preterm infants, PDA is a relatively uncommon condition that is often associated with intrinsic abnormalities of the DA and/or signalling pathways that normally trigger its closure [69,70]. In contrast, PDA in very preterm infants is mainly due to developmental immaturity. Therefore, PDA would likely not be present if the infant had been born at term [69,70].

The main limitation of our meta-analysis was the high heterogeneity. To identify the potential sources of this heterogeneity, we conducted subgroup analysis and meta-regression. The subgroup analysis focused on the various definitions of HDP, whereas the meta-regression analysed the potential moderating effect of the differences in GA between infants exposed and unexposed to HDP in each individual study. We observed that the absence of an association between HDP and the risk of developing PDA was mainly evident for preeclampsia. In contrast, a previous meta-analysis by our group showed that infants with a history of preeclampsia had higher rates of pharmacological PDA closure compared with those with a history of chorioamnionitis or growth retardation [9]. However, it is important to acknowledge that any investigation into the potential associations between the pathophysiological conditions, or endotypes, that trigger preterm birth and the outcome of prematurity is constrained by the absence of a ‘healthy’ control group. Extremely preterm birth is, by definition, a pathological condition. The healthy control would be a foetus of a similar GA that is still in the womb in physiological conditions. Furthermore, as demonstrated in the present and previous meta-analyses, preterm infants exposed to HDP tend to have a higher GA than those unexposed [9,11,12]. This results in higher clinical stability and lower incidence of respiratory complications, which may play a role in both spontaneous and pharmacologic ductal closure [9,11,12].

Low GA is the main risk factor for developing PDA [1,2,3,4]. Therefore, it is noteworthy that infants exposed to HDP, who tend to have a higher GA than unexposed infants, do not have a lower risk of developing PDA. In contrast, as evidenced by previous meta-analyses, very/extremely preterm infants exposed to HDP have a reduced risk of mortality and BPD [11,12]. At least a part of this reduced risk can be attributed to the higher GA of HDP-exposed infants [11,12]. Of note, this lower risk of mortality and BPD evolves into a higher risk when the placental dysfunction endotype is represented by growth retardation instead of HDP [11,12]. In the case of PDA, it can be speculated that the potential pathological effect of HDP on ductal development is counterbalanced or masked by the higher degree of maturation (i.e., higher GA) of the HDP-exposed infants. Thus, a higher GA may independently reduce the risk of PDA and counteract any HDP-related effects. In fact, the meta-regression analysis showed a correlation between the effect size of the association between hsPDA and BPD and the difference in GA between the HDP-exposed and control groups. However, this meta-regression is constrained by the limited number of studies that could be included. GA data for the HDP-exposed and non-exposed groups were only available in 16 studies [27,30,35,37,38,42,44,48,50,54,55,56,57,61,63]. These studies reported various outcomes, including PDA, for HDP-exposed and non-exposed infants. The remaining studies reported different risk factors for developing PDA and therefore provided GA data for infants with and without PDA.

While publication bias is a recognized issue in both randomized controlled trials and observational studies, systematic reviews have consistently shown that the risk is higher in observational research [71,72]. This is likely due to less stringent requirements for protocol registration and reporting standards in observational studies, as well as greater variability in study designs and outcomes, which can facilitate selective publication of positive findings [71,72]. Although our research question could only be addressed by observational studies, the RoBMA analysis showed no evidence in favour of the presence of publication bias in any of the meta-analyses.

Finally, the variability in the definitions of both exposures and outcomes is a substantial limitation of meta-analyses of observational studies. Despite the periodic publication of consensus documents on the classification and diagnostic criteria for HDP, there may be differences among physicians regarding the application of these criteria [73]. Furthermore, our study does not consider the degree of severity or the duration of exposure to HDP prior to birth. It is noteworthy that our previous meta-analysis demonstrated that when placental dysfunction was severe enough to induce growth retardation, there was an increased risk of developing PDA [13]. With regard to the definition of PDA, there is considerable variation in the criteria used to determine when a PDA is hemodynamically significant [4]. However, our analysis shows that the absence of an association with HDP was present for both PDA and hsPDA.

## 5. Conclusions

In conclusion, our meta-analysis shows moderate evidence for the absence of an association between HDP, particularly preeclampsia, and the risk of developing PDA. However, part of this effect may be modulated by the fact that preterm infants exposed to HDP during gestation have higher GAs. In view of the potential importance of antenatal pathological conditions for the personalised clinical care of extremely preterm newborns, it is necessary to integrate the antenatal history and placental pathology into the clinical decision-making process.

## Figures and Tables

**Table 1:** Bayesian model-averaged (BMA) meta-analysis of the association between hypertensive disorders of pregnancy and any patent ductus arteriosus in preterm infants.

Outcome	Subgroup	K	OR	95% Credible Interval		BF_10_	Evidence for		*p*-Value ^a^	BF_rf_
				Lower Limit	Upper Limit		H_1_	H_0_		
**Any PDA**	All	17	1.06	0.78	1.42	0.20		Mod.	0.514	>10^7^
	Any HDP	5	1.20	0.70	2.01	0.44		Weak	0.524	60.89
	Preeclampsia	7	0.84	0.51	1.37	0.30		Mod.	0.605	21.94
	Preeclampsia/HELLP	5	1.19	0.70	2.03	0.39		Weak	0.543	>10^3^
**Hemodynamically significant PDA**	All	25	0.89	0.74	1.08	0.27		Mod.	0.348	>10^6^
	Any HDP	8	0.84	0.62	1.12	0.45		Weak	0.316	4.56
	Preeclampsia	15	0.95	0.73	1.26	0.17		Mod.	0.401	>10^5^
	Preeclampsia/HELLP	2	0.67	0.35	1.71	1.20	Weak		0.603	2.15

^a^ *p*-value of random effects frequentist meta-analysis. BF: Bayes factor; HDP: hypertensive disorders of pregnancy; K: number of studies; Mod.: Moderate; OR: odds ratio; PDA: patent ductus arteriosus. The BF_10_ is the ratio of the probability of the data under H_1_ over the probability of the data under H_0_. Moderate evidence for H_0_ = BF_10_ from 1/10 to <1/3; weak/inconclusive evidence for H_0_ = BF_10_ from 1/3 to <1; weak/inconclusive evidence for H_1_ = BF_10_ from 1 to 3. The BF_rf_ is the ratio of the probability of the data under the random-effects model over the probability of the data under the fixed-effects model.

## Data Availability

All data relevant to the study are included in the article or uploaded as Supplementary Information.

## Supplementary Materials

The following supporting information can be downloaded at: https://www.mdpi.com/article/10.3390/children12060762/s1, Search strategy; Table S1: Characteristics of the included studies and risk of bias assessment; Table S2: Data on heterogeneity of the Bayesian model-averaged meta-analysis of the association between hypertensive disorders of pregnancy and patent ductus arteriosus in preterm infants; Table S3: Robust Bayesian meta-analysis (RoBMA) of the association between hypertensive disorders of pregnancy and patent ductus arteriosus in preterm infants; Table S4: Bayesian model-averaged meta-regression (BMA-reg) of moderating effect of the difference in GA (mean GA of HDP-exposed group minus mean GA of non-exposed group of each individual study) on the association between hypertensive disorders of pregnancy and patent ductus arteriosus in preterm infants.

## Abbreviations

The following abbreviations are used in this manuscript:

BF	Bayes factor
BMA	Bayesian model-averaged
CI	Confidence interval
CrI	Credible interval
DA	Ductus arteriosus
GA	Gestational age
HDP	Hypertensive disorders of pregnancy
H_0_	Null hypothesis
H_1_	Alternative hypothesis
OR	Odds ratio
PDA	Patent ductus arteriosus
