# Supplementary material for: Association Between Hypertensive Disorders of Pregnancy and Patent Ductus Arteriosus in Very Preterm Infants: A Bayesian Model-Averaged Meta-Analysis
Source: Children (Basel). 2025 Jun 12;12(6):762. doi: 10.3390/children12060762 (PMC12191783; doi:10.3390/children12060762)
Supplement: Supplementary file 1 [file children-12-00762-s001.zip › children-3675475-supplementary.pdf]

Supplementary materials

Association between hypertensive disorders of pregnancy and patent ductus arteriosus in very preterm infants: A Bayesian model-averaged meta-analysis.

1. Methods

1.1. Search strategy

**PubMed**<br><br>("Ductus Arteriosus, Patent"[MeSH] OR "ductus arteriosus"[Title/Abstract] OR "PDA"[Title/Abstract])<br>AND<br>(<br>("Hypertension, Pregnancy-Induced"[MeSH] OR "Pre-Eclampsia"[MeSH] OR "HELLP Syndrome"[MeSH] OR "hypertensive disorders of pregnancy" OR "preeclampsia" OR "HELLP" OR "hypertensive disorders"[Title/Abstract] OR "preeclampsia"[Title/Abstract] OR "HELLP"[Title/Abstract])<br>OR<br>("Risk Factors"[MeSH] OR "risk factors"[Title/Abstract] OR "risk factor"[Title/Abstract] OR "predictors"[Title/Abstract] OR "associated factors"[Title/Abstract] OR "prenatal risk"[Title/Abstract] OR "perinatal risk"[Title/Abstract] OR "postnatal risk"[Title/Abstract])<br>)
**Embase**<br>('patent ductus arteriosus'/exp)<br>AND<br>('pregnancy induced hypertension'/exp OR 'pre-eclampsia'/exp OR 'hypertensive disorders of pregnancy' OR 'gestational hypertension' OR 'pregnancy-induced hypertension' OR 'preeclampsia' OR 'pre-eclampsia')<br>AND<br>('infant, premature'/exp)

No language limits were set. Narrative reviews, systematic reviews, case reports, letters, editorials, and commentaries were excluded, but read to identify potential additional studies. Additional strategies to identify studies included manual review of reference lists from key articles that fulfilled our eligibility criteria, use of "related articles" feature in PubMed, and use of the "cited by" tool in Web of Science and Google scholar. Two reviewers independently screened the results of the searches, and included studies according to the inclusion criteria using EndNote (RRID:SCR_014001), using the methodology described by Bramer et al.[1].

1.2. Supplementary information on methods

Robust Bayesian meta-analysis (RoBMA)

We used RoBMA to assess the robustness of the results to the potential presence of publication bias [2]. RoBMA extends the Bayesian model-averaged meta-analysis by the two major publication bias adjustment techniques: selection models (adjusting for the publication bias operating on *p*-values) and precision-effect test and precision-effect estimate with standard errors (PET-PEESE, adjusting for the relationship between effect sizes and standard errors) [3]. The resulting RoBMA ensemble contains 36 models composed of the following assumptions about the presence vs. absence of the effect (2) x presence vs. absence of between-study heterogeneity (2) x presence vs. absence of publication bias adjustment models (6 selection models, PET, PEESE, and no bias). We used RoBMA with the same prior distributions for the effect and heterogeneity as in BMA and the default prior distributions for the publication bias adjustment part. Publication bias was expressed as BF_{bias} using the same categories for evidence previously described for BF_{10} and BF_{rf} [4].

2. Results
2.1. Supplementary Tables

Supplementary Table 1. Characteristics of the included studies and risk of bias assessment

First author. year	Country	Design	Prospective?	Total infants	Centers	Mean/median GA cohort (weeks)	Mean/median BW cohort (g)	Independent variable	Outcome	Selection	Comparability	Outcome/ Exposure	Total NOS
Ahmed 2020 [5]	Egypt	Cohort	Yes	75	1	31.4	NA	PDA	Any PDA	3	1	3	**7**
Akar 2019 [6]	Turkey	Cohort	No	389	1	29.5	1055	PDA	hsPDA	3	2	2	**7**
Bas 2014 [7]	Spain	Cohort	No	194	1	27.9	1009	PDA	hsPDA	3	1	3	**7**
Bossung 2020 [8]	Germany	Cohort	Yes	16035	62	28.3	1024	HDP	Any PDA	4	2	3	**9**
Brunner 2013 [9]	Austria	Cohort	Yes	322	1	28.5	1175	PDA	Any PDA	3	1	3	**7**
Clyman 2020 [10]	USA	Cohort	No	407	1	26.2	831	PDA	hsPDA	3	1	3	**7**
de Souza Rugolo 2015 [11]	Brasil	Cohort	Yes	60	1	26	1287	HDP	Any PDA	3	2	2	**7**
Demir 2016 [12]	Turkey	Cohort	No	235	1	29	1549	PDA	hsPDA	4	1	3	**8**
Dizdar 2012 [13]	Turkey	Ca-Co	No	361	1	28.6	1054	PDA	hsPDA	4	2	2	**8**
Echtler 2010 [14]	Germany	Cohort	No	123	1	28	1036	PDA	hsPDA	3	2	2	**7**
El-Khuffash 2008 [15]	Ireland	Cohort	Yes	80	1	28	1060	PDA	hsPDA	3	2	3	**8**
Gray 1997 [16]	Australia	Cohort	Yes	158	1	27	955	HDP	Any PDA	3	2	3	**8**
Hammoud 2003 [17]	Kuwait	Cohort	No	101	1	29.9	1094	PDA	hsPDA	3	1	3	**7**
Hentges 2015 [18]	Brazil	Cohort	Yes	88	1	29.1	1224	HDP	Any PDA	3	2	2	**7**

First author. year	Country	Design	Prospective?	Total infants	Centers	Mean/median GA cohort (weeks)	Mean/median BW cohort (g)	Independent variable	Outcome	Selection	Comparability	Outcome/ Exposure	Total NOS
Huang 2015 [19]	China	Cohort	Yes	5718	21	29.3	1161	HDP	hsPDA	4	2	3	**9**
Inayat 2015 [20]	USA	Cohort	Yes	53	1	26.6	972	PDA	hsPDA	4	2	3	**9**
Janz-Robinson 2015 [21]	Australia	Cohort	No	1473	10	26.5	945	PDA	hsPDA	4	1	3	**8**
Katheria 2018 [22]	USA	Cohort	No	292	1	28.4	1202	PDA	hsPDA	3	2	3	**8**
Kim 2018 [23]	Korea	Cohort	No	199	1	28.7	1122	HDP	Any PDA	2	2	3	**7**
Lee 2020 [24]	Korea	Cohort	No	2961	69	27.5	997	PDA	Any PDA	4	2	3	**9**
Matic 2017 [25]	Australia	Cohort	No	2549	10	26.1	914	HDP	hsPDA	4	2	3	**9**
Oliveira 2016 [26]	Portugal	Cohort	No	328	1	30	1231	PDA	Any PDA	4	1	3	**8**
Olukman 2016 [27]	Turkey	Cohort	No	824	1	29.6	1193	PDA	hsPDA	4	2	3	**9**
Patole 2007 [28]	Australia	Cohort	No	252	1	26	900	PDA	Any, hsPDA	4	2	3	**9**
Rocha 2018 [29]	Portugal	Cohort	Yes	494	11	27.6	963	HDP	hsPDA	4	2	3	**9**
Romagnoli 2018 [30]	Italy	Cohort	No	593	1	28.7	1028	PDA	Any PDA	4	2	3	**9**
Schlapbach 2010 [31]	Switzerland	Cohort	Yes	99	1	30	1130	HDP	hsPDA	4	2	3	**9**
Sellmer 2013 [32]	Denmark	Cohort	Yes	183	1	28.1	1066	PDA	Any PDA	3	1	3	**7**
Shah 2011 [33]	USA	Cohort	Yes	497	1	25.8	825	PDA	Any PDA	3	2	3	**8**
Shekharappa 2020 [34]	India	Cohort	No	88	1	30.4	1335	PDA	hsPDA	3	2	3	**8**
Soliman 2017 [35]	USA	Cohort	Yes	319	1	29	1207	HDP	Any PDA	3	2	3	**8**

First author. year	Country	Design	Prospective?	Total infants	Centers	Mean/median GA cohort (weeks)	Mean/median BW cohort (g)	Independent variable	Outcome	Selection	Comparability	Outcome/ Exposure	Total NOS
Su 2022 [36]	China	Cohort	No	956	26	27.9	911	HDP	hsPDA	4	2	3	**9**
Tokumasu 2016 [37]	Japan	Cohort	No	3218	37	25.9	758	HDP	hsPDA	4	2	3	**9**
Turunen 2011 [38]	Finland	Cohort	Yes	36	1	26.6	829	HDP	Any PDA	3	2	3	**8**
Van de Bor 1988 [39]	The Netherlands	Cohort	No	11252	101	30.5	1269.7	PDA	hsPDA	4	2	3	**9**
Velazquez 2018 [40]	USA	Cohort	No	151	1	26	800	PDA	hsPDA	3	1	3	**7**
Vieux 2010 [41]	France	Cohort	No	148	3	28.3	1114	PDA	hsPDA	3	1	3	**7**
Withagen 2000 [42]	The Netherlands	Ca-Co	No	444	1	31.3	1475	HDP	Any PDA	3	2	3	**8**
Yen 2013 [43]	Japan	Cohort	No	5727	21	29.3	1200	HDP	hsPDA	4	2	3	**9**
Yilmaz 2013 [44]	Turkey	Cohort	No	451	1	28.7	1223	HDP	Any PDA	4	2	3	**9**
Yum 2018 [45]	Korea	Cohort	Yes	71	1	27.9	1038	PDA	hsPDA	3	2	3	**8**

BW: birth weight; Ca-Co: case-control; HDP: hypertensive disorders of pregnancy; hs: hemodynamically significant; GA: gestational age; NA: not available; NOS: Newcastle-Ottawa scale, PDA: patent ductus arteriosus.

[a]Independent variable:

Supplementary Table 2. Data on heterogeneity of the Bayesian model-averaged meta-analysis of the association between hypertensive disorders of pregnancy and patent ductus arteriosus in preterm infants.

Outcome	Subgroup	K	Tau (logOR)	Credible interval		BFrf	Evidence for	
				Lower Limit	Upper Limit		Random effects	Fixed effects
Any PDA	All	17	0.51	0.30	0.82	>10^7	Extreme	
	Any HDP	5	0.51	0.19	1.15	60.89	Very strong	
	Preeclampsia	7	0.60	0.25	1.18	21.94	Strong	
	Preeclampsia/HELLP	5	0.54	0.24	1.15	>10^3	Extreme	
Hemodynamically significant PDA	All	25	0.35	0.20	0.54	>10^6	Extreme	
	Any HDP	8	0.32	0.13	0.66	4.56	Moderate	
	Preeclampsia	15	0.40	0.21	0.69	>10^5	Extreme	
	Preeclampsia/HELLP	2	0.60	0.15	1.87	2.15	Weak	

BF: Bayes factor; HDP: hypertensive disorders of pregnancy; K: number of studies; OR: odds ratio; PDA: patent ductus arteriosus.

Supplementary Table 3. Robust Bayesian meta-analysis (RoBMA) of the association between hypertensive disorders of pregnancy and patent ductus arteriosus in preterm infants.

Outcome	K	Odds ratio	Credible interval		BF_{10}	BF_{rf}	BF_{bias}
			Lower Limit	Upper Limit			
Any PDA	17	1.02	0.79	1.30	0.61	>10^5	0.49
Hemodynamically significant PDA	25	0.92	0.78	1.09	0.78	>10^6	0.33

BF Bayes factor; K: number of studies

Supplementary Table 4. Bayesian model-averaged meta-regression (BMA-reg) of moderating effect of the difference in GA (mean GA of HDP-exposed group minus mean GA of non-exposed group of each individual study) on the association between hypertensive disorders of pregnancy and patent ductus arteriosus in preterm infants.

**Prior distribution scaled to**	**Outcome**	**K**	**Intercept**		**Standardized meta-regression coefficient**		**Tau**		**BF_{10}**	**BF_{rf}**	**BF_{mod}**
			**LogOR**	**95% CrI**	**LogOR**	**95% CrI**	**LogOR**	**95% CrI**			
1/2 of the mean effect size	Any PDA	9	0.03	[-0.26, 0.34]	0.03	[-0.20, 0.26]	0.37	[0.16, 0.71]	0.46	>3x10^2	0.86
	Hemodynamically significant PDA	7	-0.21	[-0.39, -0.03]	-0.20	[-0.34, 0.02]	0.28	[0.08, 0.61]	5.54	1.04	5.75
1/4 of the mean effect size	Any PDA	9	0.03	[-0.26, 0.33]	0.01	[-0.12, 0.14]	0.37	[0.16, 0.71]	0.46	>3x10^2	0.96
	Hemodynamically significant PDA	7	-0.23	[-0.45, 0.03]	-0.08	[-0.22, 0.07]	0.31	[0.11, 0.67]	2.79	6.12	1.88

BF Bayes factor; CrI: credible interval; GA: gestational age; K: number of studies

References

1. Bramer, W.; Bain, P. Updating search strategies for systematic reviews using EndNote. *Journal of the Medical Library Association: JMLA* **2017**, *105*, 285.
2. Bartoš, F.; Maier, M.; Wagenmakers, E.J.; Doucouliagos, H.; Stanley, T. Robust Bayesian meta-analysis: Model-averaging across complementary publication bias adjustment methods. *Research Synthesis Methods* **2023**, *14*, 99-116.
3. Stanley, T.D.; Doucouliagos, H. Meta-regression approximations to reduce publication selection bias. *Research Synthesis Methods* **2014**, *5*, 60-78.
4. Lee, M.; Wagenmakers, E.-J. Bayesian data analysis for cognitive science: A practical course. **2013**.
5. Ahmed, E.G.; Samra, N.M.; Amin, S.A.; Borayek, H.A.; Abdelrazek, G. Platelets and platelet derived growth factor and ductus arteriosus in preterm neonates. *Progress in Pediatric Cardiology* **2020**, 101226.
6. Akar, S.; Topcuoglu, S.; Tuten, A.; Ozalkaya, E.; Karatepe, H.O.; Gokmen, T.; Ovali, F.; Karatekin, G. Is the First Postnatal Platelet Mass as an Indicator of Patent Ductus Arteriosus? *Archives of Iranian Medicine* **2019**, *22*, 687.
7. Bas-Suárez, M.P.; González-Luis, G.E.; Saavedra, P.; Villamor, E. Platelet counts in the first seven days of life and patent ductus arteriosus in preterm very low-birth-weight infants. *Neonatology* **2014**, *106*, 188-194.
8. Bossung, V.; Fortmann, M.I.; Fusch, C.; Rausch, T.; Herting, E.; Swoboda, I.; Rody, A.; Härtel, C.; Göpel, W.; Humberg, A. Neonatal outcome after preeclampsia and HELLP syndrome: a population-based cohort study in germany. *Frontiers in Pediatrics* **2020**, *8*, 579293.
9. Brunner, B.; Hoeck, M.; Schermer, E.; Streif, W.; Kiechl-Kohlendorfer, U. Patent ductus arteriosus, low platelets, cyclooxygenase inhibitors, and intraventricular hemorrhage in very low birth weight preterm infants. *J Pediatr* **2013**, *163*, 23-28, doi:10.1016/j.jpeds.2012.12.035.
10. Clyman, R.I.; Hills, N.K. The effect of prolonged tracheal intubation on the association between patent ductus arteriosus and bronchopulmonary dysplasia (grades 2 and 3). *Journal of Perinatology* **2020**, *40*, 1358-1365.
11. de Souza Rugolo, L.M.S.; de Sá, M.P.A.; Kurokawa, C.S.; Madoglio, R.J.; Bentlin, M.R.; Jr, A.R.; Corrente, J.E. There is no difference in nitric oxide metabolites and neonatal outcome between premature infants born to pre-eclamptic and those born to normotensive women. *Paediatrics and International Child Health* **2015**, *35*, 47-52.
12. Demir, N.; Peker, E.; Ece, İ.; Ağengin, K.; Bulan, K.A.; Tuncer, O. Is platelet mass a more significant indicator than platelet count of closure of patent ductus arteriosus? *The Journal of Maternal-Fetal & Neonatal Medicine* **2016**, *29*, 1915-1918.
13. Dizdar, A.E.; Ozdemir, R.; Nur Sari, F.; Yurttutan, S.; Gokmen, T.; Erdeve, O.; Emre Canpolat, F.; Uras, N.; Suna Oguz, S.; Dilmen, U. Low platelet count is associated with ductus arteriosus patency in preterm newborns. *Early Hum Dev* **2012**, *88*, 813-816, doi:10.1016/j.earlhumdev.2012.05.007.
14. Echtler, K.; Stark, K.; Lorenz, M.; Kerstan, S.; Walch, A.; Jennen, L.; Rudelius, M.; Seidl, S.; Kremmer, E.; Emambokus, N.R.; et al. Platelets contribute to postnatal occlusion of the ductus arteriosus. *Nat Med* **2010**, *16*, 75-82, doi:10.1038/nm.2060.
15. El-Khuffash, A.F.; Molloy, E.J. Influence of a patent ductus arteriosus on cardiac troponin T levels in preterm infants. *J Pediatr* **2008**, *153*, 350-353, doi:10.1016/j.jpeds.2008.04.014.
16. Gray, P.H.; Hurley, T.M.; Rogers, Y.M.; O'Callaghan, M.J.; Tudehope, D.I.; Burns, Y.R.; Phty, M.; Mohay, H.A. Survival and neonatal and neurodevelopmental outcome of 24–29 week gestation infants according to primary cause of preterm delivery. *Australian and New Zealand journal of obstetrics and gynaecology* **1997**, *37*, 161-168.
17. Hammoud, M.S.; Elsori, H.A.; Hanafi, E.A.; Shalabi, A.A.; Fouda, I.A.; Devarajan, L.V. Incidence and risk factors associated with the patency of ductus arteriosus in preterm infants with respiratory distress syndrome in Kuwait. *Saudi Med J* **2003**, *24*, 982-985.
18. Hentges, C.R.; Silveira, R.C.; Procianoy, R.S. Angiogenic and antiangiogenic factors in preterm neonates born to mothers with and without preeclampsia. *American Journal of Perinatology* **2015**, *32*, 1185-1190.
19. Huang, H.-C.; Yang, H.-I.; Chou, H.-C.; Chen, C.-Y.; Hsieh, W.-S.; Tsou, K.-I.; Tsao, P.-N.; Group, T.P.I.D.C.S. Preeclampsia and retinopathy of prematurity in very-low-birth-weight infants: a population-based study. *PloS one* **2015**, *10*, e0143248.

20. Inayat, M.; Bany-Mohammed, F.; Valencia, A.; Tay, C.; Jacinto, J.; Aranda, J.V.; Beharry, K.D. Antioxidants and biomarkers of oxidative stress in preterm infants with symptomatic patent ductus arteriosus. *American Journal of Perinatology* **2015**, *32*, 895-904.
21. Janz-Robinson, E.M.; Badawi, N.; Walker, K.; Bajuk, B.; Abdel-Latif, M.E.; Bowen, J.; Sedgley, S.; Carlisle, H.; Smith, J.; Craven, P. Neurodevelopmental outcomes of premature infants treated for patent ductus arteriosus: a population-based cohort study. *The Journal of pediatrics* **2015**, *167*, 1025-1032. e1023.
22. Katheria, V.; Poeltler, D.; Brown, M.; Hassen, K.; Patel, D.; Rich, W.; Finer, N.; Katheria, A. Early prediction of a significant patent ductus arteriosus in infants< 32 weeks gestational age. *Journal of Neonatal-Perinatal Medicine* **2018**, *11*, 265-271.
23. Kim, D.-H.; Shin, S.H.; Kim, E.-K.; Kim, H.-S. Association of increased cord blood soluble endoglin with the development of bronchopulmonary dysplasia in preterm infants with maternal preeclampsia. *Pregnancy Hypertension* **2018**, *13*, 148-153.
24. Lee, J.A.; Sohn, J.A.; Oh, S.; Choi, B.M. Perinatal risk factors of symptomatic preterm patent ductus arteriosus and secondary ligation. *Pediatrics & Neonatology* **2020**, *61*, 439-446.
25. Matić, M.; Inati, V.; Abdel-Latif, M.E.; Kent, A.L.; Network, N.A.N. Maternal hypertensive disorders are associated with increased use of respiratory support but not chronic lung disease or poorer neurodevelopmental outcomes in preterm neonates at< 29 weeks of gestation. *Journal of Paediatrics and Child Health* **2017**, *53*, 391-398.
26. Oliveira, A.; Soares, P.; Flor-de-Lima, F.; Neves, A.L.s.; Guimarães, H.l. PDA management in VLBW infants: experience of a level III NICU. *Journal of Pediatric and Neonatal Individualized Medicine (JPNIM)* **2016**, *5*, e050227-e050227.
27. Olukman, O.; Ozdemir, R.; Karadeniz, C.; Calkavur, S.; Mese, T.; Vergin, C. Is there a relationship between platelet parameters and patency of ductus arteriosus in preterm infants? *Blood Coagulation & Fibrinolysis* **2017**, *28*, 8-13.
28. Patole, S.K.; Kumaran, V.; Travadi, J.N.; Brooks, J.M.; Doherty, D.A. Does patent ductus arteriosus affect feed tolerance in preterm neonates? *Archives of Disease in Childhood-Fetal and Neonatal Edition* **2007**, *92*, F53-F55.
29. Rocha, G.; de Lima, F.F.; Machado, A.P.; Guimaraes, H. Preeclampsia predicts higher incidence of bronchopulmonary dysplasia. *Journal of Perinatology* **2018**, *38*, 1165-1173, doi:10.1038/s41372-018-0133-8.
30. Romagnoli, V.; Pedini, A.; Santoni, M.; Scutti, G.; Colaneri, M.; Pozzi, M.; Cogo, P.E.; Carnielli, V.P. Patent ductus arteriosus in preterm infants born before 30 weeks' gestation: high rate of spontaneous closure after hospital discharge. *Cardiology in the Young* **2018**, *28*, 995-1000.
31. Schlapbach, L.J.; Ersch, J.; Adams, M.; Bernet, V.; Bucher, H.U.; Latal, B. Impact of chorioamnionitis and preeclampsia on neurodevelopmental outcome in preterm infants below 32 weeks gestational age. *Acta paediatrica* **2010**, *99*, 1504-1509.
32. Sellmer, A.; Bjerre, J.V.; Schmidt, M.R.; McNamara, P.J.; Hjortdal, V.E.; Høst, B.; Bech, B.H.; Henriksen, T.B. Morbidity and mortality in preterm neonates with patent ductus arteriosus on day 3. *Archives of Disease in Childhood-Fetal and Neonatal Edition* **2013**, *98*, F505-F510.
33. Shah, N.A.; Hills, N.K.; Waleh, N.; McCurnin, D.; Seidner, S.; Chemtob, S.; Clyman, R. Relationship between circulating platelet counts and ductus arteriosus patency after indomethacin treatment. *The Journal of pediatrics* **2011**, *158*, 919-923. e912.
34. Shekharappa, C.B.; Elizabeth, E.A.B.; Balachander, B. Association of patent ductus arteriosus size with clinical features and short-term outcomes in preterm infants less than 34 weeks. *Indian Journal of Child Health* **2020**, 105-108.
35. Soliman, N.; Chaput, K.; Alshaikh, B.; Yusuf, K. Preeclampsia and the risk of bronchopulmonary dysplasia in preterm infants less than 32 weeks' gestation. *American journal of perinatology* **2017**, *34*, 585-592.
36. Su, Z.; Huang, W.; Meng, Q.; Jia, C.; Shi, B.; Fan, X.; Cui, Q.; Chen, J.; Wu, F. Mothers with hypertensive disorders of pregnancy increased risk of periventricular leukomalacia in extremely preterm or extremely low birth weight infants: A propensity score analysis. *Frontiers in Pediatrics* **2022**, *10*, 978373.
37. Tokumasu, H.; Tokumasu, S.; Kawakami, K. Impact of pre-eclampsia in extremely premature infants: Population-based study. *Pediatrics International* **2016**, *58*, 578-583, doi:10.1111/ped.12905.
38. Turunen, R.; Andersson, S.; Laivuori, H.; Kajantie, E.; Siitonen, S.; Repo, H.; Nupponen, I. Increased postnatal inflammation in mechanically ventilated preterm infants born to mothers with early-onset preeclampsia. *Neonatology* **2011**, *100*, 241-247.

39. van de Bor, M.; Verloove-Vanhorick, S.P.; Brand, R.; Ruys, J.H. Patent ductus arteriosus in a cohort of 1338 preterm infants: a collaborative studya. *Paediatric and perinatal epidemiology* **1988**, *2*, 328-336.
40. Velazquez, D.M.; Reidy, K.J.; Sharma, M.; Kim, M.; Vega, M.; Havranek, T. The effect of hemodynamically significant patent ductus arteriosus on acute kidney injury and systemic hypertension in extremely low gestational age newborns. *The Journal of Maternal-Fetal & Neonatal Medicine* **2019**, *32*, 3209-3214.
41. Vieux, R.; Desandes, R.; Boubred, F.; Semama, D.; Guillemin, F.; Buchweiller, M.-C.; Fresson, J.; Hascoet, J.-M. Ibuprofen in very preterm infants impairs renal function for the first month of life. *Pediatric Nephrology* **2010**, *25*, 267-274.
42. Withagen, M.I.; Visser, W.; Wallenburg, H.C. Neonatal outcome of temporizing treatment in early-onset preeclampsia. *European Journal of Obstetrics & Gynecology and Reproductive Biology* **2001**, *94*, 211-215.
43. Yen, T.-A.; Yang, H.-I.; Hsieh, W.-S.; Chou, H.-C.; Chen, C.-Y.; Tsou, K.-I.; Tsao, P.-N.; Group, T.P.I.D.C.S. Preeclampsia and the risk of bronchopulmonary dysplasia in VLBW infants: a population based study. *PloS one* **2013**, *8*, e75168.
44. Yılmaz, Y.; Kutman, H.G.K.; Ulu, H.Ö.; Canpolat, F.E.; Uraş, N.; Oğuz, S.S.; Dilmen, U. Preeclampsia is an independent risk factor for spontaneous intestinal perforation in very preterm infants. *The Journal of Maternal-Fetal & Neonatal Medicine* **2014**, *27*, 1248-1251.
45. Yum, S.K.; Moon, C.-J.; Youn, Y.-A.; Lee, J.Y.; Sung, I.K. Echocardiographic assessment of patent ductus arteriosus in very low birthweight infants over time: prospective observational study. *The Journal of Maternal-Fetal & Neonatal Medicine* **2018**, *31*, 164-172.
